# Emphysematous Cystitis With Intestinal Pseudo-Obstruction in a 72-Year-Old Woman: A Multidisciplinary Case Report

**DOI:** 10.7759/cureus.88347

**Published:** 2025-07-20

**Authors:** Stanislaw Szymkiewicz

**Affiliations:** 1 Department of Urology, Janusz Korczak Provincial Specialist Hospital in Słupsk, Słupsk, POL

**Keywords:** bladder wall gas, emphysematous cystitis, escherichia coli (e. coli), intestinal pseudo-obstruction, urinary tract infection

## Abstract

Emphysematous cystitis (EC) is a rare, potentially life-threatening urinary tract infection characterized by gas formation within the bladder wall and lumen, typically associated with diabetes, immunosuppression, or advanced age. We present the case of a 72-year-old woman with systemic sclerosis and hypothyroidism, who was admitted for an infected distal limb wound following femoropopliteal bypass. During hospitalization, she developed emphysematous cystitis accompanied by radiological features of intestinal pseudo-obstruction. Prompt diagnosis, conservative treatment with bladder catheterization and intravenous antibiotics, and multidisciplinary management resulted in clinical improvement. This case highlights the importance of early imaging and collaborative care in atypical urinary tract infections.

## Introduction

Emphysematous cystitis (EC) is a rare form of complicated urinary tract infection caused by gas-forming microorganisms, most commonly *Escherichia coli* [1]. Risk factors include advanced age, diabetes mellitus, immunosuppression, and structural or functional urinary tract abnormalities [2]. The clinical presentation may be subtle and nonspecific, making imaging, particularly computed tomography (CT), crucial for diagnosis [3]. It is important to distinguish emphysematous pyelonephritis (EPN) as a related but distinct entity: unlike EC, which involves gas within the bladder wall and lumen, EPN is a severe necrotizing infection of the renal parenchyma caused by gas-forming bacteria such as *Escherichia coli* and *Klebsiella pneumoniae*. EPN is associated with higher morbidity and mortality than EC and often requires more aggressive intervention, including possible surgical drainage or nephrectomy. This distinction highlights the need for accurate imaging and differential diagnosis in patients presenting with atypical urinary tract infections. Despite its potentially severe course, the early identification of EC often allows for successful conservative management [4]. Emphysematous cystitis occurring in patients with autoimmune conditions, such as systemic sclerosis, has been rarely described in the literature, underscoring the novelty and clinical relevance of this case.

## Case presentation

**Figure 1:**
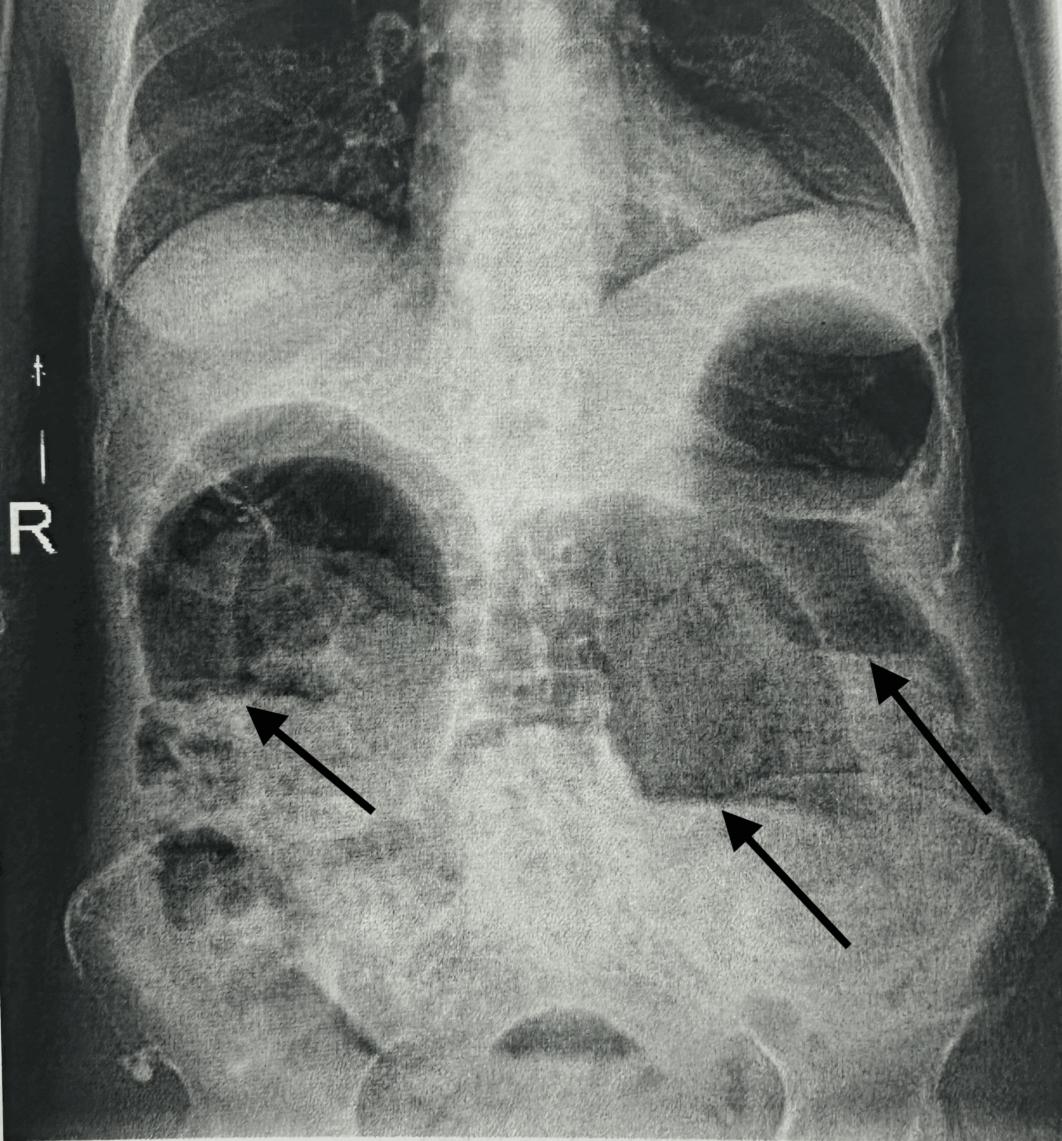
Abdominal radiograph demonstrates multiple centrally located, dilated bowel loops with visible air-fluid levels. Visible air-fluid levels indicated by arrows.

**Figure 2:**
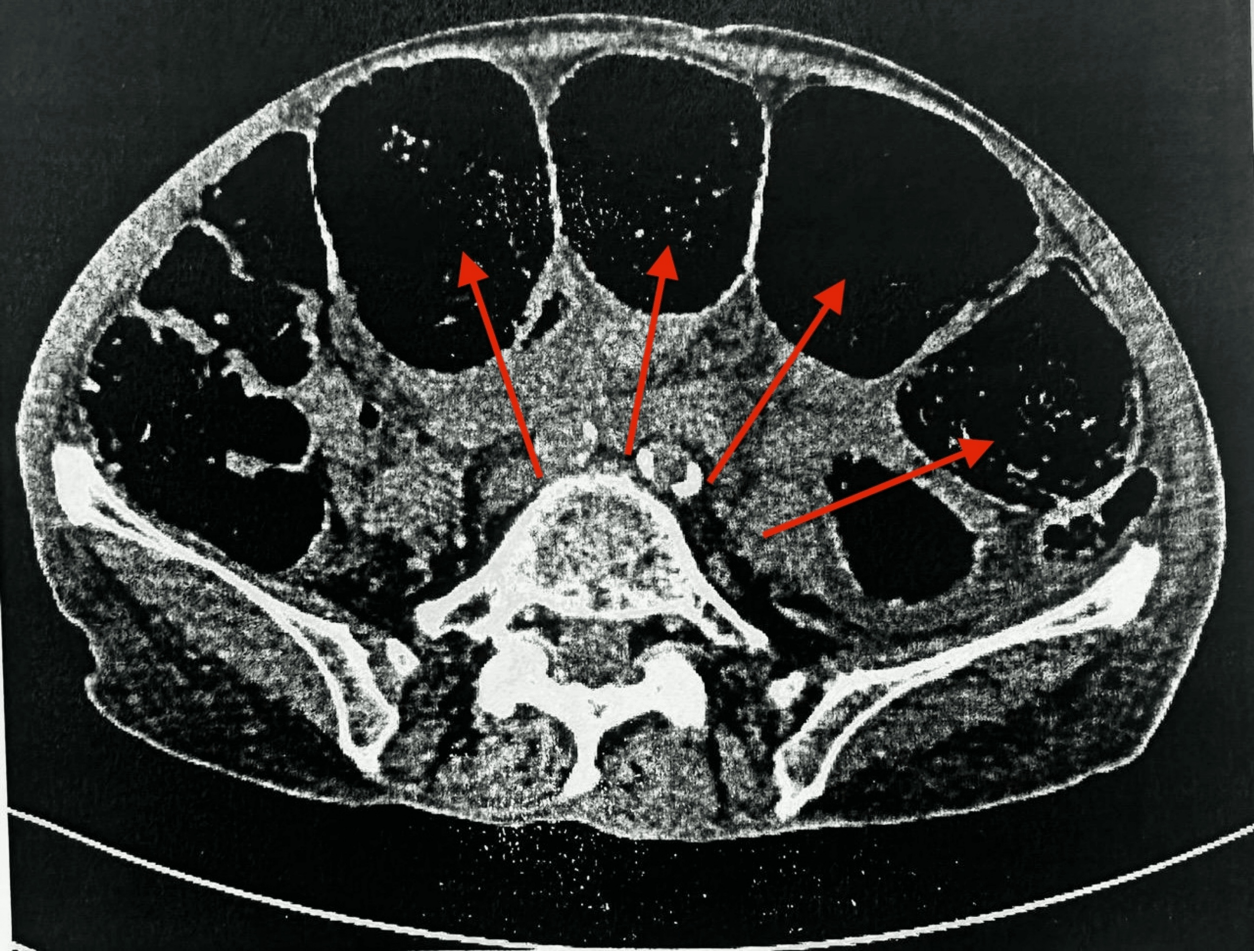
CT scan showing the marked gaseous distension of large bowel loops up to ~10 cm, consistent with large bowel obstruction. Arrows indicate distended bowel loops. CT: computed tomography

**Figure 3:**
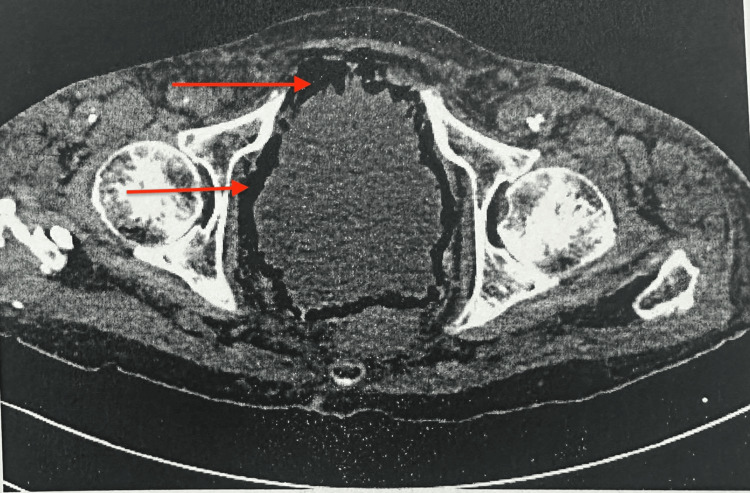
Axial CT at the pelvic level. The bladder is markedly distended, with intramural gas along the anterior and lateral wall, suggestive of emphysematous cystitis. Arrows indicate intramural gas. CT: computed tomography

A 72-year-old woman with a history of systemic sclerosis, hypothyroidism, atherosclerosis, and active smoking was admitted to the vascular surgery department in May 2025 due to an infected distal wound following femoropopliteal bypass (FP3). The patient had a fever (38°C), mild lower abdominal discomfort, and constipation but no urinary urgency, frequency, dysuria, or hematuria. Due to signs of systemic infection and the clinical suspicion of intestinal obstruction, a plain abdominal X-ray was performed, revealing fecal retention and dilated bowel loops up to 10 cm in diameter with air-fluid levels (Figure 1). These findings were consistent with features of bowel pseudo-obstruction. The initial differential diagnoses included bowel obstruction, intra-abdominal abscess, and urinary tract infection. Sepsis was suspected based on elevated inflammatory markers and systemic signs such as fever and mild abdominal distension. Empirical broad-spectrum intravenous antibiotics (ceftriaxone) were initiated along with fluid resuscitation and supportive care while awaiting culture results. The pseudo-obstruction was managed conservatively with bowel rest, nasogastric decompression, and electrolyte correction. A contrast-enhanced CT scan of the abdomen and pelvis confirmed the marked gaseous distension of the bowel loops (Figure 2), as well as findings of emphysematous cystitis: the urinary bladder was markedly distended with urine, showing intramural and perivesical gas, particularly along the anterior bladder wall, suggestive of necrosis or microperforation (Figure 3). There was no evidence of free peritoneal air. Initial laboratory tests showed elevated inflammatory markers but no other significant abnormalities. The results are presented in Table 1.

**Table 1:** Initial laboratory results with reference ranges. CRP, C-reactive protein; GFR, glomerular filtration rate

Parameter	Result	Reference range
CRP	80.1 mg/L	0-5 mg/L
Procalcitonin	0.24 ng/mL	<0.5 ng/mL
Leukocytes	8.91 × 10⁹/L	4-10 × 10⁹/L
Creatinine	0.71 mg/dL	0.6-1.3 mg/dL
GFR	85 mL/minute/1.73 m²	>60 mL/minute/1.73 m²
Sodium	136 mmol/L	135-145 mmol/L
Potassium	3.3 mmol/L	3.5-5.1 mmol/L
Albumin	3.4 g/dL	3.5-5.2 g/dL
Total protein	5.3 g/dL	6.4-8.3 g/dL

**Figure 4:**
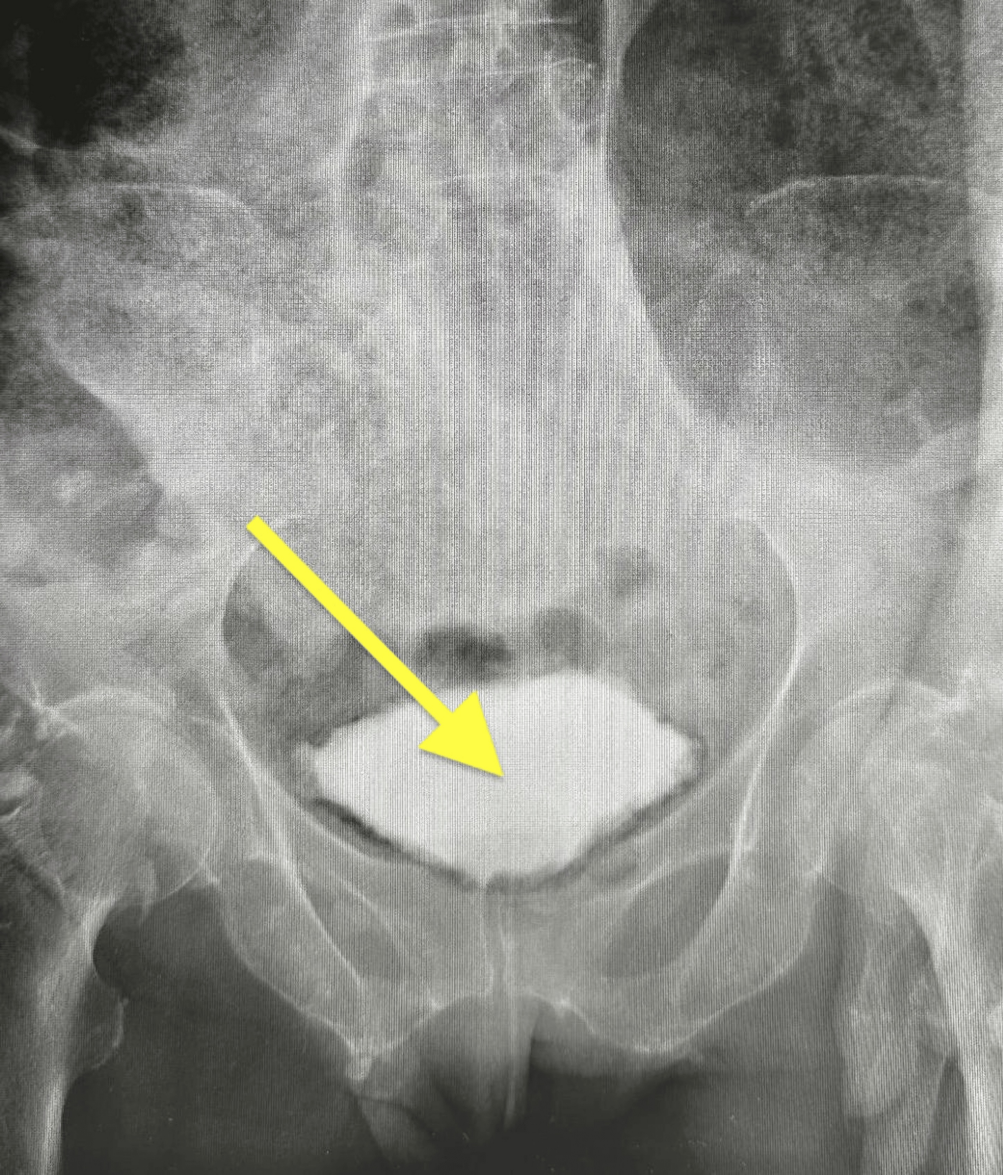
Cystourethrography, oblique view. Contrast-filled bladder without extravasation. Bladder wall irregularity visible. The arrow indicates contrast material within the urinary bladder.

**Figure 5:**
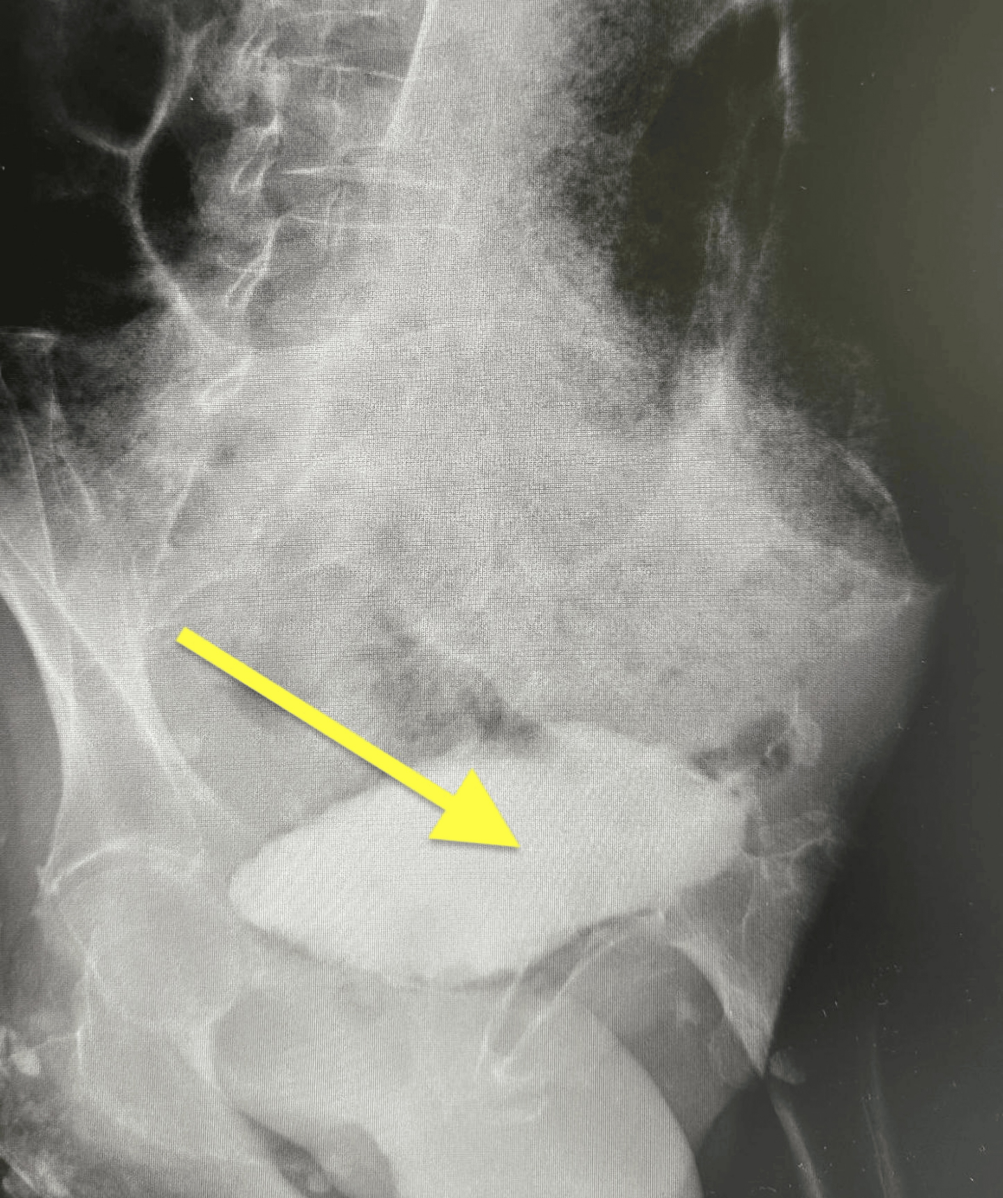
Cystourethrography, AP view. No evidence of perforation. The arrow indicates contrast material within the urinary bladder. AP: anteroposterior

Urological consultation recommended bladder catheterization, empirical antibiotic therapy with ceftriaxone (which was continued after culture confirmation due to sensitivity), and urine/blood cultures. Bladder catheterization was performed without resistance. Approximately 800 mL of cloudy urine was drained; the urine was sent for culture and microscopy. Meropenem and clindamycin were initially considered due to the severity of the presentation and the need for broader anaerobic coverage but ultimately were not required once cultures confirmed sensitivity to ceftriaxone. Follow-up cystourethrography revealed no extravasation of contrast but confirmed bladder wall irregularities and intramural gas (Figure 4). A control image in the anteroposterior view excluded any bladder perforation (Figure 5). Urine culture grew *Escherichia coli* (extended-spectrum beta-lactamase {ESBL}-negative), which was resistant to trimethoprim/sulfamethoxazole, fluoroquinolones, ampicillin, and amoxicillin/clavulanate but susceptible to ceftriaxone and other beta-lactams. Intravenous ceftriaxone was continued for 10 days, followed by an additional five days of oral antibiotics (cefixime) to complete the course, based on culture sensitivity. The patient’s clinical condition improved steadily with conservative management. She remained afebrile, and her abdominal distension resolved within several days. Routine laboratory tests showed a gradual decrease in inflammatory markers, with the normalization of C-reactive protein (CRP) and procalcitonin within two weeks. Follow-up urine cultures were sterile. A follow-up CT scan confirmed the regression of intramural gas and the resolution of bladder wall irregularities, with no evidence of new complications. The radiological improvement correlated well with the patient’s sustained clinical recovery. The bladder catheter was removed after adequate drainage, and no further urinary retention or recurrent infection occurred.

## Discussion

Emphysematous cystitis (EC) is a rare but potentially serious form of complicated urinary tract infection characterized by gas accumulation within the bladder wall, usually resulting from the bacterial fermentation of glucose or protein substrates [1,2]. This gas-forming infection is typically caused by organisms such as *Escherichia coli* or *Klebsiella pneumoniae* [1] and most often affects elderly women with comorbidities [2]. Diabetes mellitus remains the most frequently reported risk factor [2,5], but other conditions, such as systemic sclerosis, atherosclerosis, and advanced age, can also predispose to EC due to impaired immunity and altered tissue perfusion [2,6].

Systemic immune dysregulation and the use of immunosuppressive agents such as corticosteroids or disease-modifying antirheumatic drugs (DMARDs) may further increase vulnerability to EC [2,6]. However, even in the absence of these medications, patients with autoimmune diseases such as systemic sclerosis may remain at heightened risk. As highlighted by Murata et al., emphysematous cystitis can occasionally be complicated by pneumoperitoneum without full-thickness bladder rupture, mimicking gastrointestinal emergencies [7].

The clinical presentation of EC can be subtle and nonspecific, with symptoms ranging from dysuria and suprapubic pain to systemic signs of infection [2,3]. In rare cases, it may present with minimal or no urinary symptoms, leading to delays in diagnosis. In our case, a severe systemic inflammatory response contributed to secondary bowel pseudo-obstruction, which has also been described in the literature as a rare complication of pelvic infections [1,2].

Imaging is crucial in establishing the diagnosis. While plain radiographs may detect intravesical gas, contrast-enhanced computed tomography (CT) remains the gold standard due to its ability to localize gas within the bladder wall, rule out bladder rupture, and assess associated complications such as perivesical inflammation or necrosis [1,2,5]. In our patient, CT imaging confirmed emphysematous cystitis and associated ileus, while cystourethrography ruled out frank perforation.

The management of EC is typically conservative and includes bladder drainage with a Foley catheter, empirical intravenous antibiotics adjusted based on culture results, and supportive care [2,5]. In the present case, ceftriaxone was used empirically and proved effective, with subsequent clinical and radiological improvement. Surgical intervention is usually reserved for patients with bladder necrosis, perforation, or progressive sepsis despite optimal medical therapy [2,5]. González Flores et al. also highlighted that the cornerstone of successful management is timely bladder decompression and tailored antimicrobial therapy, even in atypical presentations [8].

We reviewed similar reports in the literature. Kang et al. described a case where EC mimicked intestinal perforation, ultimately managed conservatively [1]. Ranjan et al. reviewed 113 cases and emphasized the role of early imaging in avoiding unnecessary surgical procedures [2]. Stein et al. reported EC complicated by iliopsoas abscess and pneumorrhachis [3], while Ertürk and Yıldırım presented a case with bilateral hydroureteronephrosis [4]. Thomas et al. provided a broader perspective through a review of 135 cases, reaffirming CT as the key diagnostic modality [5]. Otero-Colón et al. [6] and Choi et al. [9] further discussed the importance of recognizing EC in nondiabetic and immunocompromised populations. Lastly, Tan et al. demonstrated the wide variability in clinical course and outcomes among EC patients, from incidental findings to severe infections [10].

In conclusion, emphysematous cystitis remains a diagnostic and therapeutic challenge, especially in elderly or immunocompromised individuals. This case reinforces the importance of early CT imaging, prompt bladder decompression, and individualized antibiotic treatment. A high index of suspicion, particularly in atypical or asymptomatic presentations, is essential for optimal patient outcomes.

## Conclusions

This case demonstrates that not just individuals with diabetes are at risk of emphysematous cystitis; patients with other immunosuppressive conditions, such as systemic sclerosis, or advanced age should also be monitored for signs of complicated urinary tract infections. A high level of clinical suspicion is essential, especially when symptoms are subtle or atypical. Timely imaging and culture-based antibiotic therapy can help avoid unnecessary surgical interventions and reduce morbidity. Clinicians should remember that early recognition and conservative management remain possible and effective, even in complex cases.
